# Supplementary material for: A modern method of multiple working hypotheses to improve inference in ecology
Source: R Soc Open Sci. 2020 Jun 3;7(6):200231. doi: 10.1098/rsos.200231 (PMC7353960; doi:10.1098/rsos.200231)
Supplement: Simulating multiple hypotheses about species distributions. [file rsos200231supp1.pdf]

Supplement 1: Simulating multiple hypotheses about species distributions.

Package Installation

The `checkyourself` package is available for free on github: https://github.com/syanco/checkyourself. Users can download the code directly from that source (or use git to clone the entire repository). Downloading or cloning the repository would make the source code available to users for customization.

The package can also be installed as an R library directly from within the R environment. Installation as a package would let the user replicate the code presented below, as well as to modify the parameterizations and model run specifics (e.g., iterations). The easiest way to install the package from within R is to use the `install_github` function from the `devtools` package. `devtools` can be installed with a call to `install.packages('devtools')`. Alternatively, users can run the code below, which will check whether `devtools` is already installed and, if it is not, will install it.

```
if(!("devtools" %in% installed.packages()[,"Package"])){
  install.packages("devtools")
} else {
  print("devtools is already installed!")
}
```

Once `devtools` is installed, the `checkyourself` package can be installed by running the following code:

```
devtools::install_github("syanco/checkyourself")
```

Introduction

This supplement uses a simple individual-based simulation model of animals settling a landscape to consider multiple competing hypotheses about processes that give rise to species distributions. For this example, we simulate a patchy landscape consisting of two habitat types ("Habitat A" and "Habitat B"). We consider three competing hypotheses about how a population of animals may settle that landscape:

1. **Null Model:** Individuals settle the landscape randomly with no influence of habitat or neighbors.
2. **Habitat Preference (HP) Model:** Individuals settle the landscape preferring "Habitat A" over "Habitat B".
3. **Conspecific Attraction (CA) Model:** Individuals settle the landscape preferring to settle near already-settled locations.

The fundamental question imagined for this example is whether these distinct settlement processes produce distinct patterns in distribution. Put differently, can the observation of a given distribution pattern, relative to the underlying habitat, imply a particular settlement process. Many species distribution models assume that disproportionate use of certain habitats necessarily implies a preference or requirement for that habitat. We here consider whether alternative hypothetical settlement processes can lead to distributions that converge with those generated by habitat preference.

Many of the functions included in the `checkyourself` package are called by the top-level functions found in this supplement. Therefore, in this document we only describe how to run the models in their simplest forms. However, we encourage readers interested in modifying, expanding, or evaluating these models to view and modify the code directly; the vignettes associated with the package provide additional detail about the structure of the code and the objects returned by the various functions.

Simulating a Patchy Landscape

The first step is to load the `checkyourself` package.

```
library(checkyourself)
```

We now create a set of simulated landscapes on which the simulations can run. To do this, we use the function `simlandscapes`, the arguments of which define the nature of the landscape as well as the relative degree of habitat preference to be exhibited in the habitat preference simulations (this degree of preference is "baked into" the landscape but can be ignored by the null and conspecific attraction models). Below we create a 100 x 100 cell landscape with approximately 50 patches of Habitat A, approximately 100 cells in size each. We also create versions of this landscape with different strengths of preference coded as relative factors of preference for Habitat A over Habitat B. For this example we consider 5 parameterizations of the Habitat Preference Hypothesis with preference strengths (`A.coef`) of 1.5, 2.0, 2.5, 3.0, and 3.5.

```
#declare parameterizations for habitat preference models (must be done as part
#of landscape generation process).
A.coef <- c(1.5, 2, 2.5, 3, 3.5)
#simulate a patchy landscape
lands <- simlandscapes(A.coef = A.coef, #supply preference strengths
                       matsize = 100, #set landscape size
                       #set approximate target number of patches of Habitat A
                       n.clusters = 50,
                       #set approximate target size of each patch of Habitat A
                       size.clusters = 100)
```

Run Simulations

We are now ready to simulate the multiple parameterizations of the three models on this simulated landscape. We first establish the declarations which parameterize the models and determine the number of individual agents included in each model as well as the number of iterations of each model parameterization to run. Note that we have already declared the parameterization for the HP Model as part of the landscape generation process (`A.coef`). The Conspecific Attraction model simulates a settlement processes whereby the first individual settles randomly (with no preference for Habitat A or B) and subsequent individuals choose their settlement location from within some radius of previously settled individuals. The magnitude of that radius can be adjusted as a parameter in the model to modify the strength of conspecific attraction (e.g., small radii lead to strong conspecific attraction by forcing each settling individual to choose locations very near to individuals already on the landscape)

```
reps <- 100 #number of iterations to run of each parameterization
n.individ <- 100 #number of animals to simulate settling in each iteration
radius <- c(1,3,5,10,25) #define settlment radii for conspecific attraction
```

Null Model

The Null Model can be run with a single call to `repRand`. We supply `repRand` with several of the parameterizations from above. We also need to supply the function with one of the habitat matrices in the object `lands` as well as the corresponding `A.coef` so that it can identify which cells represent Habitat A. While the simulation itself does not utilize this habitat matrix, because part of the output reports the proportion of settled location within Habitat A, one of the habitat matrices is required. It does not matter which one since they are identical in their spatial geometry and the magnitude of the preference encoded is not considered. The only requirement is that the habitat matrix supplied must match the value of `A.coef` supplied, since that value serves as the "key" to which cells are part of Habitat A. Remember that the habitat matrices are

stored in the second dimension of the habitat array, which is, itself, the first element of the list `lands`. Below we simply use the first habitat matrix and the first value of `A.coef`.

```
NULLmod <- repRand(reps=reps, #number of iterations of each parameterization
                   n.individ = n.individ, #number of individuals to simulate
                   hab.mat = lands[[1]][,1], #any habitat matrix
                   #value of A.coef mtaching hab.mat as a "key"
                   A.coef = A.coef[1])
```

Habitat Preference Model

The HP Model is run by a call to `repHab`. This function takes all the same arguments as `rep.rand` plus the probability matrices contained in the second element of `lands` (which define the probability of any cell being selected for settlement, based on the strength of preference for each habitat). Note that for this model we must supply all the habitat matrices and values of `A.coef` so that the model can iterate through each parameterization.

```
HABmod <- repHab(p.mat=lands[[2]], #the probability matrix
                 hab.mat = lands[[1]],  #the habitat preference matrix
                 reps = reps, #number of iterations of each parameterization
                 n.individ = n.individ, #number of individuals so simulate
                 A.coef = A.coef) #list of habitat preference parameterizations
```

Conspecific Attraction Model

The CA Model is run with a call to `repCon`. This model again requires one of the habitat matrices contained in the first element of `lands` and the corresponding value from `A.coef` as was the case for the Null Model. In addition to the `reps` and `n.individ` required by all the models, this function also requires the vector of parameterizations for the CA Model contained in `radius`. Finally, this function requires that we supply a matrix with cell values corresponding to cell IDs in the argument `ID.mat`. This matrix was simulated as part of the patchy landscape and is contained in the third element of `lands`.

```
#NOTE - THIS CODE WILL TAKE A SUBSTANTIAL AMOUNT OF TIME TO RUN
CAmod <- repCon(reps = reps, #number of iterations of each parameterization
                radius = radius, #list of CA parameterizations
                ID.mat = lands[[3]], #matrix cell ID matrix
                hab.mat = lands[[1]][,1], #any habitat matrix
                n.individ = n.individ, #number of individuals so simulate
                #value of A.coef mtaching hab.mat as a "key"
                A.coef = A.coef[1])
```

Analysis and Visualization

We can pull the ranges of the sampling distributions for each model parametrization with the function `compilerangesSDM`.

```
simdat <- compilerangesSDM(nulldata = NULLmod, #Null Model simulated data
                           habdata = HABmod, #HP Model simulated data
                           condata= CAmod, #CA Model simluated data
                           A.coef = A.coef, #HP Model parameterizations
                           radius = radius) #CA Model parameterizations
```

```r
#manually set the factor order
simdat$parameter <- factor(simdat$parameter,
                           levels = c("--", "1", "1.5", "2", "2.5", "3", "3.5",
                                      "5", "10", "25"))
```

Whisker Plot of Sampling Distribution Ranges

We'll first produce a whiskers plot showing the range of the sampling distributions produced by the simulations arranged by parameterization. This is a useful plot for estimating overlap between outcomes, as well as the overall amount of variance produced by each simulation and structures in variance across parameterizations.

```r
library(ggplot2)
#> Warning: package 'ggplot2' was built under R version 3.5.3
whiskers <- ggplot(data=simdat, aes(xmin = low, xmax = upp,
                                    y = parameter)) +
  geom_errorbarh() +
  facet_grid(model ~ ., scales = "free_y", space = "free_y") +
  xlab("Proportion Habitat A") +
  ylab("Parameter Value") +
  ggtitle("Sampling Distribution Ranges") +
  theme(axis.ticks.y = element_blank(), axis.title = element_text(size=8),
        plot.title = element_text(hjust = 0.5),
        plot.margin = margin(t = 0, r = 0, b = 0.2, l = 0, unit = "in"),
        panel.background = element_rect(fill= "white", color = "black"),
        panel.grid.major = element_blank(), panel.grid.minor = element_blank())
whiskers
```

Heat Map of Sampling Distribution Overlap

Next we can produce a heat map showing the showing the proportion of overlap between model-parameterization combinations (1 represents complete overlap, 0 represents no overlap). Overlap entails specifying some interval based on one distribution (in this case the range, but could be 95% CIs, etc.) and calculating the quantiles of the other distribution that are contained within that interval.

In order to compare sampling distributions to each other to search for degenerate relationships, we calculate the unidirectional pairwise overlap between all sampling distributions. Each overlap was unidirectional since different model parameterizations produced unequal variances. Thus, the overlap between any two sampling distributions may be (and *is* in this case) asymmetric. We combined all unidirectional pairwise comparisons into matrices of overlap between each combination of models (each matrix including all the simulated parameterizations). We then rearrange the data into long format so that we can subsequently call graphing functions from ggplot2.

```r
#get the pairwise unidirectional combinations
modelcomb <- getcombos(X = list("RAND"=NULLmod, "HP"=HABmod, "CA"=CAmod),
                       Y = list("RAND"=NULLmod, "HP"=HABmod, "CA"=CAmod))

#produce overlap matrices for each combination
heats <- vectorheat(modelcomb[[1]], modelcomb[[2]])

#change data structure to long form to comport with ggplot
heatsmelt <- meltheatmats(heats)

#plot will look nicer if we manually reset the factor order for the CA model...
```

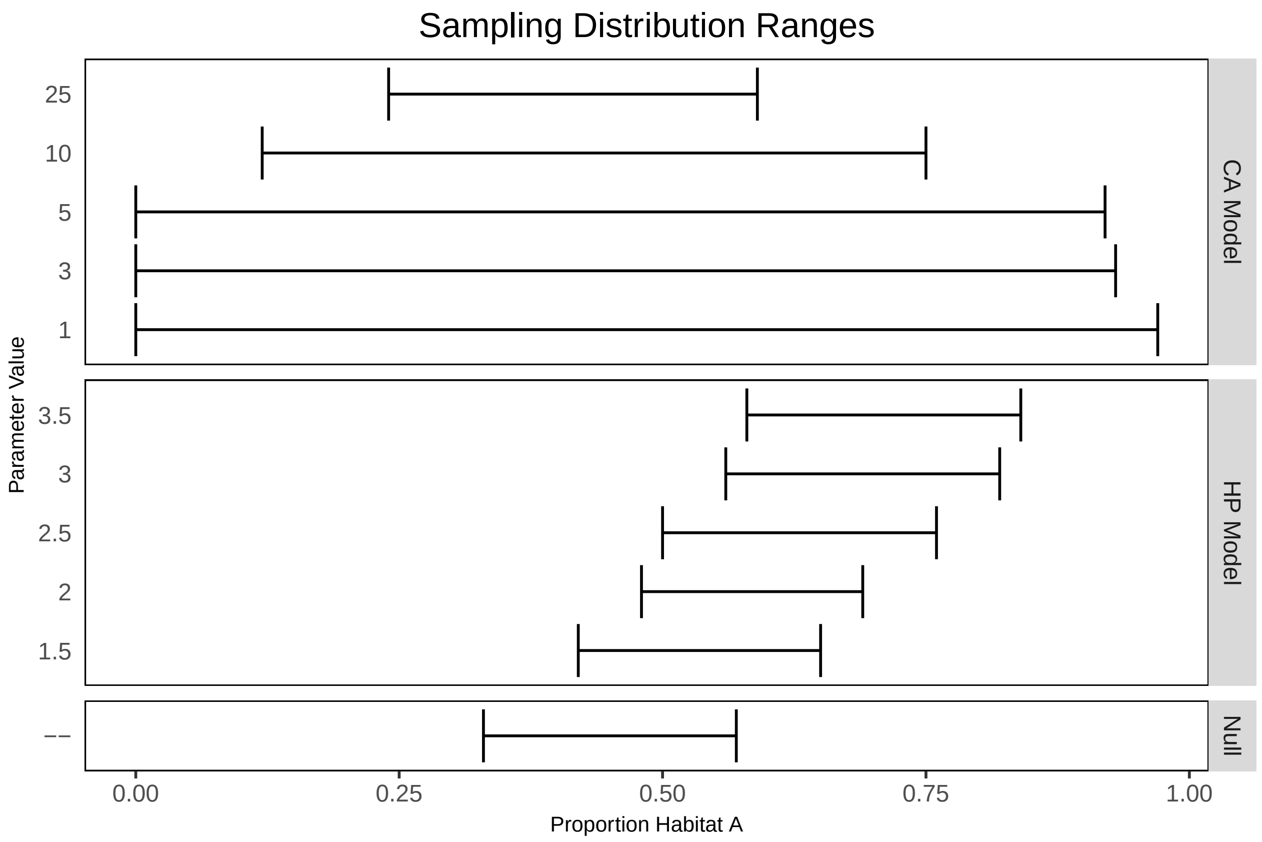

Figure 1: Whisker plot of sampling distribution ranges

```r
heatsmelt[[3]]$CA <- factor(heatsmelt[[3]]$CA,
                            levels = c("1", "3", "5", "10", "25"))
heatsmelt[[6]]$CA <- factor(heatsmelt[[6]]$CA,
                            levels = c("1", "3", "5", "10", "25"))
heatsmelt[[7]]$CA <- factor(heatsmelt[[7]]$CA,
                            levels = c("1", "3", "5", "10", "25"))
heatsmelt[[8]]$CA <- factor(heatsmelt[[8]]$CA,
                            levels = c("1", "3", "5", "10", "25"))
heatsmelt[[9]]$CA <- factor(heatsmelt[[9]]$CA,
                            levels = c("1", "3", "5", "10", "25"))
```

Now we can visualize the degree of overlap in sampling distributions for each unidirectional pairwise model combination (and for each parameterization of those models)

```r
#create the component plots
heatmaplist <- plotheatvector(heatsmelt)

#remove the plots that compare a model with itself
heatmaplist <- heatmaplist[!sapply(heatmaplist, is.null)]

#arrange the plots on a grid and display
gridExtra::grid.arrange(grobs = heatmaplist,
                        #layout matrix puts the plots in a clearer order
                        layout_matrix = rbind(c(1,6,2), c(3,4,5)))
```

Interpretation

Noisy Hypotheses

In order to examine the variances produced by each model, compare variances between models, and examine how variance relates to parameterization, we calculated the range for each sampling distribution produced by the 11 model-parameterization combinations. We plotted these together to visually assess model-generated variances relative to other model-parameterizations and to assess parameter related structure between models.

In this example we can see several interesting patterns that a researcher engaged in this analysis could pursue. The strongest conspecific attraction models (lowest values of radius) produce high variances, likely due to the strong effect of the first individual to settle in the patchy landscape. As conspecific attraction gets weaker the values and variance become comparable to the null model. There is also clear structure in the values estimated by the habitat preference models: we observed a higher proportion of "Habitat A" selected by models with stronger habitat preference, as would be expected. Variance was relatively constant between models suggesting that parameter estimation under this hypothesis would be similarly accurate regardless of the magnitude of the parameter estimate itself.

Note that the range is not the only way the variance could be explored; 95% highest density intervals, variance, standard deviation (for normal distributions), or interquartile range are all suitable methods for exploring the variance generated by a model. The choice of metric for examining spread in the simulated sampling distributions should be made by the researcher taking into account the nature of planned inferential methods, biological relevance of each measurement, and distributional assumptions about the expected response variable(s).

Whether a hypothesis is "too noisy" is subject to the judgement of the researcher and the goals of the study. For example, a study aimed at estimating some parameter should consider whether the predicted uncertainty around that estimate (measured from the simulated sampling distributions of that parameter) is sufficient for

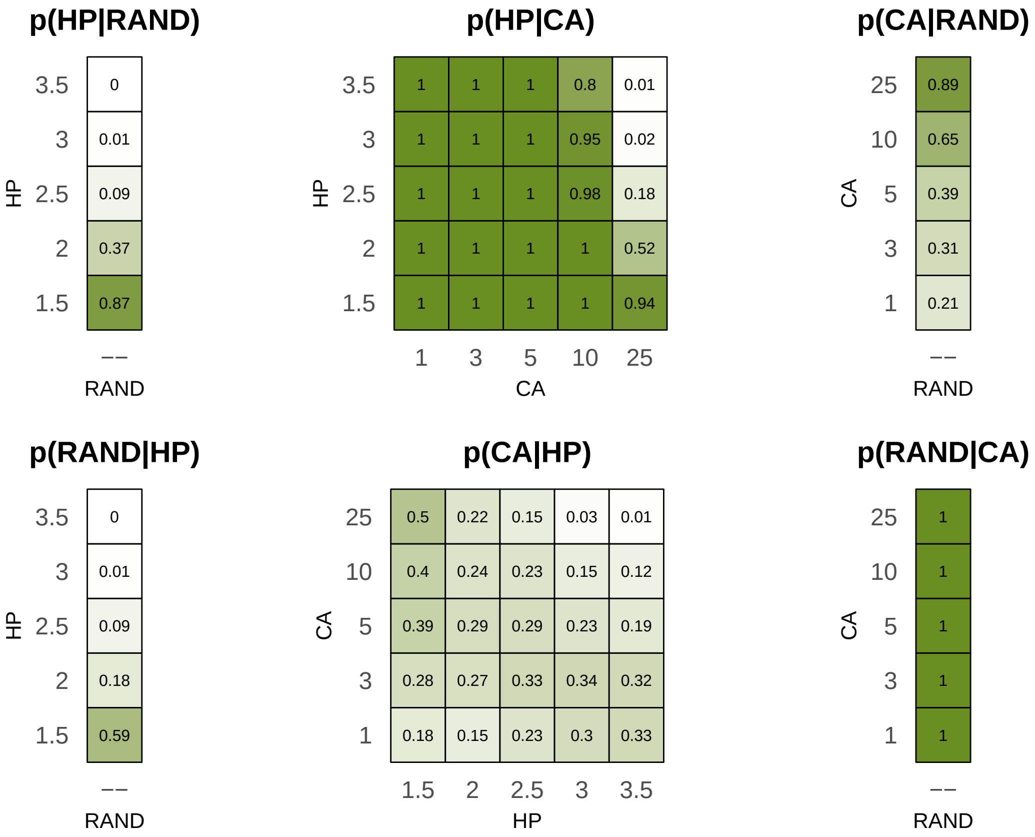

Figure 2: Heatmap of sampling distribution overlap. Panels clockwise from top-left: p(HP|RAND) shows the proportion of habitat preference model simulations that overlapped the range of null models for each parameterization; p(HP|CA) shows the proportion of habitat preference model simulations that overlapped the range of conspecific attraction models for each parameterization; p(CA|RAND) shows the proportion of conspecific attraction model simulations that overlapped the range of null models for each parameterization; p(RAND|CA) shows the proportion of null model simulations that overlapped the range of conspecific attraction models for each parameterization;p(CA|HP)shows the proportion of conspecific attraction model simulations that overlapped the range of habitat preference models for each parameterization; p(RAND|HP) shows the proportion of null model simulations that overlapped the range of habitat preference models for each parameterization.

the study's aims. Researchers may also use these analyses to consider the modality and distributional form of the simulated sampling distribution.

Hypothesis Degeneration

In order to compare sampling distributions to each other to search for degenerate relationships, we calculated the unidirectional pairwise overlap between all sampling distributions. Each overlap was unidirectional since different model parameterizations produced unequal variances. Thus, the overlap between any two sampling distributions was asymmetric. We combined all unidirectional pairwise comparisons into heatmaps to assess patterns of overlap in parameter combinations.

We observed clear structures in the degeneracy of certain model-parameterization combinations. For example, the proportion of habitat preferences models overlapped by conspecific attraction models was very high for models with low strength of preference and/or strong conspecific attraction (small radius). Conversely, the proportion of conspecific attraction models that overlapped habitat preference models was generally low except for models with very strong habitat preference and strong conspecific attraction. It is important to note here that because of the unequal variances these patterns are not the inverse of each other.

Ultimately, as with noisy hypotheses, determining if sampling distributions overlap "too much" is up to the judgement of the researcher within the context of the study's aims. Each unidirectional pairwise proportion of overlap represents the conditional probability of one hypothesis generating response data capable of being produced by another hypothesis. For example, the overlap described by p(HP|Null) is the probability of the habitat preference hypothesis producing convergent results given the null hypothesis. Note that this does not represent the overall probability of mis-specifying a hypothesis as any other because each overlap statement is a probability conditioned on the assumption of a particular model.

Revising Hypotheses

Given both the large variance generated for the null model and the high amount of overlap in sampling distributions between several model-parameterization combinations, it's reasonable to assume that a researcher in this situation would seek to refine their proposed study. There are myriad options for such revision and in a "real world" examination this would rest on the judgement and system-specific knowledge of the researcher as well as the specific aims of the study. We offer a few potential revisions here to illustrate the types of changes that could be made but in no way suggest that these revisions are exhaustive or appropriate to the system.

By including some spatial measures as part of the observed response pattern, models that converged when considering only proportion of habitat selected may now be parsed. In many instances, the various model-parameterization combinations produced differentiable spatial patterns. For example, many of the models that hypothesized conspecific attraction exhibited strong spatial clustering, likely resulting from the strong influence of the initially settled location.

```r
#create a vector of one of the habitat maps as a factor varibale
base <- factor(lands[[1]][,3], levels = c("2.5", "1"))

#create a raster object from one of the habitat matrices
land <- raster::raster(matrix(lands[[1]][,3], #supply the 3rd habitat matrix
                              #get the side dimensions of the matrix supplied
                              nrow = sqrt(length(lands[[1]][,3])),
                              byrow = T)) #fill by row

#set the extent to match cell-based numbering
raster::extent(land) <- c(1, 100, 1, 100)
```

```r
#GET COORDINATES OF A SINGLE CA MODEL RUN
#get cell values from a single model iteration
cells.con <- unlist(CAmod[[1]][1])

#create matrix from which to draw coordinates
mat <- matrix(1:length(lands[[1]][,3]), nrow = sqrt(length(lands[[1]][,3])))

#convert cell values into coordinates
coords.con <- arrayInd(mat[cells.con], .dim = dim(mat))

#set column names
colnames(coords.con) <- c("x_coord", "y_coord")


#GET COORDINATES OF A SINGLE HP MODEL RUN
#get cell values from a single model iteration
cells.hab <- unlist(HABmod[[1]][1])

#convert cell values into coordinates
coords.hab <- arrayInd(mat[cells.hab], .dim = dim(mat))

#set column names
colnames(coords.hab) <- c("x_coord", "y_coord")

#PLOT THE EXAMPLE COORDINATES
library(rasterVis)
#> Loading required package: raster
#> Warning: package 'raster' was built under R version 3.5.3
#> Loading required package: sp
#> Loading required package: lattice
#> Loading required package: latticeExtra
#> Loading required package: RColorBrewer
#>
#> Attaching package: 'latticeExtra'
#> The following object is masked from 'package:ggplot2':
#>
#>     layer

#make a map for the CA Model coordinates
map1 <- gplot(land) + #basemap showing the habitat patches
  geom_raster(aes(fill = factor(value))) +
  coord_equal() + #
  scale_fill_manual(values = c("#41AB5D", "#FFEDA0"), labels = c("B", "A")) +
  #add the CA Model coordinates
  geom_point(data = as.data.frame(coords.con),
             aes(x = x_coord, y = y_coord),
             fill = "#2B8CBE", size = 1.5, shape = 21, color = "black",
             stroke = 1) +
  guides(fill=F) +
  ggtitle("CA Model (Radius = 3)") +
  theme(axis.ticks = element_blank(), axis.text = element_blank(),
        axis.title = element_blank(),
        plot.margin = margin(t = .2, r = 0, b = 0.0, l = 0, unit = "in"),
```

```r
        plot.title = element_text(size = 11, face = "bold", hjust = 0.5),
        panel.background = element_rect(fill= "white", color = "black"),
        panel.grid.major = element_blank(), panel.grid.minor = element_blank())

#make a map for the HP Model coordinates
map2 <- gplot(land) + #basemap showing the habitat patches
  geom_raster(aes(fill = factor(value))) +
  coord_equal() +
  scale_fill_manual(values = c("#41AB5D", "#FFEDA0"), labels = c("B", "A")) +
  #add the CA Model coordinates
  geom_point(data = as.data.frame(coords.hab),
             aes(x = x_coord, y = y_coord), fill = "#2B8CBE",
             size = 1.5, shape = 21, color = "black", stroke = 1)+
  guides(fill=guide_legend(title="Habitat Type", reverse = T,
                           direction = "horizontal")) +
  ggtitle("HP Model (Pref. Strength = 2)") +
  theme(axis.ticks = element_blank(), axis.text = element_blank(),
        axis.title = element_blank(),
        plot.margin = margin(t = .2, r = 0, b = 0.0, l = 0, unit = "in"),
        plot.title = element_text(size = 11, face = "bold", hjust = 0.5),
        panel.background = element_rect(fill= "white", color = "black"),
        panel.grid.major = element_blank(), panel.grid.minor = element_blank())

#make a common legend
g_legend<-function(a.gplot){
  tmp <- ggplot_gtable(ggplot_build(a.gplot))
  leg <- which(sapply(tmp$grobs, function(x) x$name) == "guide-box")
  legend <- tmp$grobs[[leg]]
  return(legend)}

mylegend<-g_legend(map2)

#plot the maps together
gridExtra::grid.arrange(gridExtra::arrangeGrob(map1, map2 +
                                                 theme(legend.position="none"),
                                               nrow = 1, padding = 0),
                        mylegend, nrow=2, heights = c(3,1), padding = 0)
```

Manipulative experimentation might also help to parse the convergent hypotheses. Decoy experiments have been used previously as both a management tool for recolonization efforts and a test of conspecific attraction (Kress 1983, Kotliar and Burger 1984, Ward et al. 2011). Similarly, habitat manipulation could also be used to parse convergent hypotheses (e.g., Cruz-Angón et al. 2008).

Addressing the model-parameterization combinations that are producing very high levels of variance might not be so easily accomplished through variable refinement or manipulative experimentation. Because the simulation model assumes no observation error, additional processes or poorly constrained processes are the likely culprits. Indeed, in reconsidering the conspecific attraction model we can see that the resulting spatial distribution is a combination of two separate processes: 1) the initial individual settles randomly; and 2) subsequent individuals settle based on the conspecific attraction decisions rules.

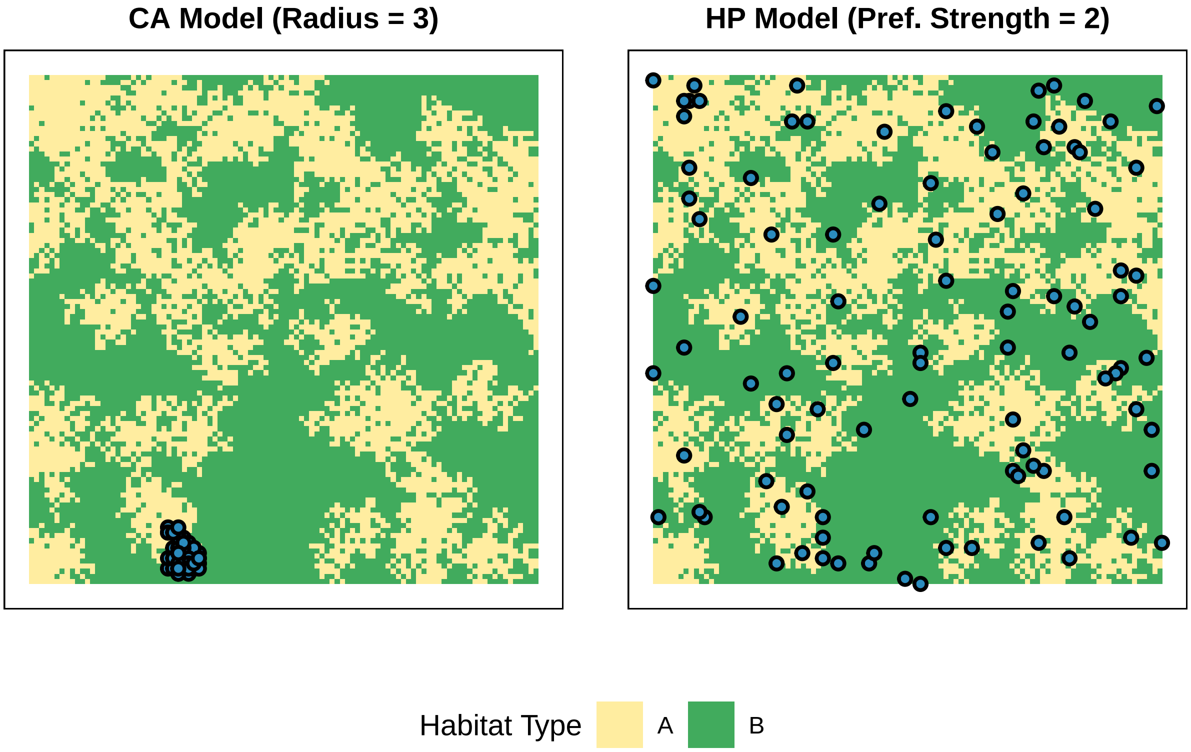

Figure 3: Map of settled distributions
