# Supplementary material for: A modern method of multiple working hypotheses to improve inference in ecology
Source: R Soc Open Sci. 2020 Jun 3;7(6):200231. doi: 10.1098/rsos.200231 (PMC7353960; doi:10.1098/rsos.200231)
Supplement: Simulating multiple hypotheses about animal habitat selection [file rsos200231supp2.pdf]

Supplement 2: Simulating multiple hypotheses about animal habitat selection

Package Installation

The `checkyourself` package is available for free on github: https://github.com/syanco/checkyourself. Users can download the code directly from that source (or use git to clone the entire repository). Downloading or cloning the repository would make the source code available to users for customization.

The package can also be installed as an R library directly from within the R environment. Installation as a package would let the user replicate the code presented below, as well as to modify the parameterizations and model run specifics (e.g., iterations). The easiest way to install the package from within R is to use the `install_github` function from the `devtools` package. `devtools` can be installed with a call to `install.packages('devtools')`. Alternatively, users can run the code below, which will check whether `devtools` is already installed and, if it is not, will install it.

```
if(!("devtools" %in% installed.packages()[,"Package"])){
  install.packages("devtools")
} else {
  print("devtools is already installed!")
}
```

Once `devtools` is installed, the `checkyourself` package can be installed by running the following code:

```
devtools::install_github("syanco/checkyourself")
```

Introduction

This supplement uses the hypothesis vetting process to evaluate multiple hypotheses about animal movement and habitat selection processes. The simulation will use simple individual-based models included in the `checkyourself` package. We simulate two movement processes: 1) movement with a preference for a particular habitat; and 2) movement that is random with respect to habitat. We use hypothesis vetting to understand the unique identifiability of each of these hypotheses based on the response variable of habitat preference. We note at the outset that this is a reduced hypothesis set and we advise a more robust consideration of multiple hypotheses; we restrict our intial hypothesis set in this example to illustrate the effect of unconsidered processes as will become clear.

Many of the functions included in the `checkyourself` package are called by the top-level functions found in this supplement. Therefore, in this document we only describe how to run the models in their simplest forms. However, we encourage readers interested in modifying, expanding, or evaluating these models to view and modify the code directly; the vignettes associated with the package provide additional detail about the structure of the code and the objects returned by the various functions.

Simulate a Patchy Landscape

The first step is to load the `checkyourself` package.

```
library(checkyourself)
set.seed(3) #set the seed so results will be reproducible
```

We need a patchy landscape on which the simulation can run. We can use the same `simlandscapes` function described in Supplement 1. Just as with that example, the relative strength of habitat preference (parameter) for "Habitat A" over "Habitat B" is set at this stage and "baked into" the landscape. (Note that the degree to which this preference mixes with other drivers of movement, such as limiting the distance of each move or return to a central place, is set later.) For simplicity, in this simulation, we will only consider a single value for `A.coef` - where any patch of Habitat A is twice as likely to be chosen, all else being equal, than a given patch of Habitat A.

```
matsize <- 100 #set size of matrix (one side of square matrix in cells)

#strength of preference for "Habitat A" (as factor relative to "Habitat B")
A.coef <- 2.5

#simulate a patchy landscape
lands <- simlandscapes(A.coef = A.coef, #supply preference strengths
                        matsize = matsize, #set landscape size
                        #set approximate target number of patches of Habitat A
                        n.clusters = 25,
                        #set approximate target size of each patch of Habitat A
                        size.clusters = 200)
```

Run Simulations

Now we run the movement model on this simulated landscape. In this step of the hypothesis vetting process we are comparing the competing hypotheses of 1) random movements (with respect to habitat); and, 2) preference for Habitat A. We simulate a central-place forager who must make return visits to a, e.g., nest or den on probabilistically defined intervals. Both scenarios include a term to implement a preference for shorter versus longer steps. In the first scenario, the habitat preference has 10 times the weight of the preference to make shorter steps; in the second scenario, the habitat preference is turned off entirely.

```
steps <- 100 #how many movements should each agent make
pd.rate <- .5 #probability of returning to the central place on each step
#coefficient of the exponential dist. describing tendency to short movements
lambda <- .01
coef.d <- 1 #initial relative weighting of distance parameter
coef.r <- 10 #initial relative weighting of habitat preference parameter

#define number of iterations for generating the sampling distribution for each
#hypothesis/parameterization.
#NOTE: THIS CODE WILL TAKE A SUBSTANTIAL AMOUNT OF TIME TO RUN
iter <- 100

move.iter.hab <- replicate(iter, chooseLoc(hab.prob = as.numeric(lands[[2]]),
                                            pd.rate = pd.rate, steps = steps,
                                            lambda = lambda, coef.d = coef.d,
                                            coef.r = coef.r, matsize = matsize),
                            simplify = F)

coef.r <- 0 #reduce habitat preference to zero

move.iter.np <- replicate(iter, chooseLoc(hab.prob = as.numeric(lands[[2]]),
                                           pd.rate = pd.rate, steps = steps,
                                           lambda = lambda, coef.d = coef.d,
```

```
                        coef.r = coef.r, matsize = matsize),
                simplify = F)
```

Analysis and Visualization

We now compare the simulated sampling distributions. We first convert the raw locations into the response variable of interest. Commonly in animal movement studies, preference for some resource is of primary interest. Here we will asses preference for Habitat A, given the relative availability of that habitat in the underlying landscape There are nearly countless metrics available for assessing habitat preference at an individual scale (see e.g., Manly 2002) - for simplicity's' sake, we will use perhaps the simplest formulation of a preference metric: the foraging ratio of Savage (1931). Note that the specific movement/preference question being considered may dictate a different response variable be used by other researchers. This is especially the case in situations where the definition of availability is non-trivial. However, we leave that question as specific to movement ecologists and here proceed with a simple and intuitive metric for habitat preference in order to demonstrate the hypothesis vetting process.

Foraging Ratio

The foraging ratio (w_i) is simply the proportional use of a given habitat by an animal (o_i) relative to its proportional availability (π_i):

$w_i = o_i/\pi_i$

where,

$o_i = u_i/u_+$

with u_i representing use of habitat i and u_+ representing the total use of all habitats; and, similarly,

$\pi_i = a_i/a_+$ with a_i representing the total amount of habitat i available and a_+ representing the total population of available habitat.

Putting this all together:

$w_i = \frac{o_i}{\pi_i} = \frac{u_i/u_+}{a_i/a_+}$

We can easily get the proportional availability of a given habitat (in this case we'll simply consider the preference for Habitat A) from the cell counts in the simulated landscape:

```
pi_A <- sum(lands[[1]] == A.coef)/matsize^2
```

In order to calculate the proportion used we can use the `getPropUsed` function in the `checkyourself` package. Further, we can iterate this function across the results of each iteration of the simulation to collect the simulated sampling distribution of proportional use.

```
o_i.hab <- sapply(move.iter.hab, getPropUsed, hab.mat = lands[[1]],
            A.coef = A.coef, matsize = matsize)

o_i.np <- sapply(move.iter.np, getPropUsed, hab.mat = lands[[1]],
            A.coef = A.coef, matsize = matsize)
```

The final step to convert these sampling distributions to the strength of *preference* (rather than use) is to divide by the `pi_A` that we previously calculated. This metric can take values from $0 - \infty$; a value of 1 would indicate habitat use that is perfectly in proportion with availability whereas values below or above 1 suggest under- or over-selection, respectively. Note that no null hypothesis test is used here, though there are other

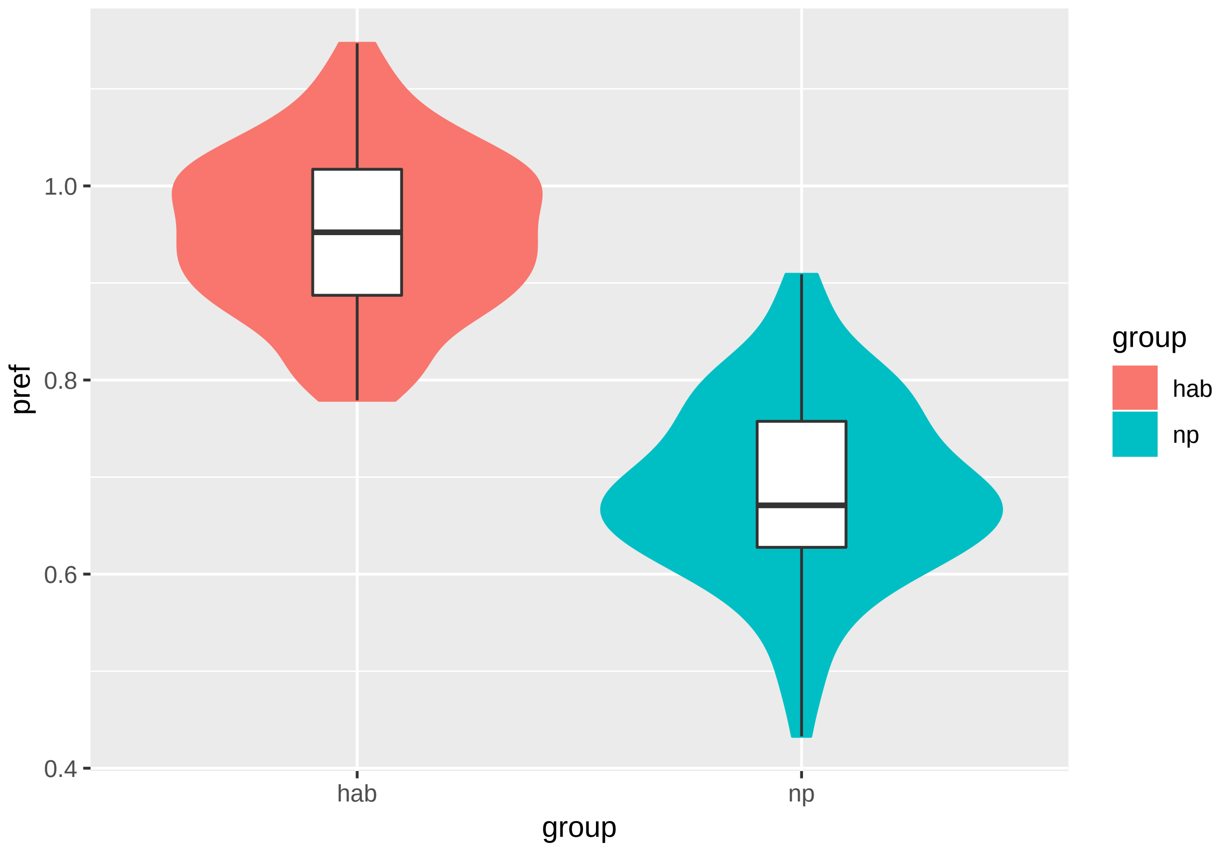

Figure 1: Sampling distributions of habitat preference between models

constructions that would so allow, if desired; the point here is to understand the characteristics of these sampling distributions.

```
w_i.hab <- o_i.hab/pi_A

w_i.np <- o_i.np/pi_A

sim_data <- rbind(data.frame("pref" = w_i.hab,
                             "group" = rep("hab", length(w_i.hab))),
                  data.frame("pref" = w_i.np,
                             "group" = rep("np", length(w_i.np))))
```

One way to visualize these sampling distributions is via violin plots with associated box plots:

```
library(ggplot2)
```

```
## Warning: package 'ggplot2' was built under R version 3.5.3
```

```
ggplot(sim_data) +
  geom_violin(aes(y=pref, x = group, color = group, fill = group)) +
  geom_boxplot(aes(y = pref, x = group), width = .2)
```

Interpretation

We can clearly see some convergence between the two competing hypotheses - both sampling distributions overlap to a potentially problematic degree. Simply estimating visually, we can see that >50% of the Habitat Preference Model sampling distribution is within the range of the No Preference Model. The habitat preference model is also producing many simulations with no apparent preference (foraging ratio values close to 1), or even a slight *avoidance* of Habitat A. Note that this overlap/noisiness is only for a single parameterization of habitat preference (wherein Habitat A is 2.5X as likely to be selected as Habitat B and this prefernce is 10 times stronger than the tendency for short step lengths). We might expect the overlap to shrink with increasing strength of prefernce and/or relative weigthing with other movement drivers and an interested researcher could explore additional parameterizations. As is, this relatively strong simulated habitat preference shows large overlap with a process containing no habitat preference whatsover. Moreover, the no preference model is showing many iterations with apparent *avoidance* of Habitat A. A researcher wishing to disentangle this might consider different metrics of habitat preference (e.g. step selection functions that consider availability of habitat at the scale of the step, not the individual) or behavior switching models that allow for the segregation of movements given assumptions about behavioral modes (e.g., perhaps removing those steps that return to the central place would provide greater distinction, since that type of movement is a a fundamentally different behavioral process which we would not expect to affect habitat preference).

Model Revision

Building on the final point from the section above, we here filter the locations at the central place to remove the process of returning to the central place and then repeat the calculation of foraging ratio and the visualization of the sampling distributions.

We pause to point out that this essentially means we had initially failed to consider a relevant hypothesis that contributes to the observed pattern in resource selection. Our approach here is to now censor those portions of the repsonse variable that are driven by that new process, but we could just as easily build a more complex analytic model that allowed for behavior switching. This is a case where the analyses of only two candidate hypotheses revealed that set to be insufficient and our coding of the model included additional unconsidered sources of variance.

```
#Filter Habitat Preference Model
move.iter.hab.filt <- sapply(move.iter.hab, FUN = function(x, matsize) {
  keep <- sapply(x, FUN = function(x, matsize) {
    if(x[1] != floor(matsize/2) && x[2] != floor(matsize/2)) {
      keep <- x
    } else {
      keep <- NA
    }
    return(keep)
  }, matsize = matsize, simplify = T)
  keep <- keep[!is.na(keep)]
  return(keep)
}, matsize = matsize, simplify = F)

#Filter No Preference Model
move.iter.np.filt <- sapply(move.iter.np, FUN = function(x, matsize) {
  keep <- sapply(x, FUN = function(x, matsize) {
    if(x[1] != floor(matsize/2) && x[2] != floor(matsize/2)) {
      keep <- x
    } else {
      keep <- NA
```

```
    }
    return(keep)
  }, matsize = matsize, simplify = T)
  keep <- keep[!is.na(keep)]
  return(keep)
}, matsize = matsize, simplify = F)

#calculate proportional use for each model
o_i.hab.filt <- sapply(move.iter.hab.filt, getPropUsed, hab.mat = lands[[1]],
            A.coef = A.coef, matsize = matsize)
o_i.np.filt <- sapply(move.iter.np.filt, getPropUsed, hab.mat = lands[[1]],
            A.coef = A.coef, matsize = matsize)

#calculate foraging ratio for each model
w_i.hab.filt <- o_i.hab.filt/pi_A
w_i.np.filt <- o_i.np.filt/pi_A

#put it all in a data.frame
sim_data.filt <- rbind(data.frame("pref" = w_i.hab.filt,
                              "group" = rep("hab", length(w_i.hab.filt))),
                  data.frame("pref" = w_i.np.filt,
                              "group" = rep("np", length(w_i.np.filt))))

library(ggplot2)
#produce violin plot
ggplot(sim_data.filt) +
  geom_violin(aes(y=pref, x = group, color = group, fill = group)) +
  geom_boxplot(aes(y = pref, x = group), width = .2)
```

Reinterpretation

Clearly the process of returning to the nest was producing additional noise and driving some convergence between the two hypotheses. By revisting the construction of our model and accounting for additional processes in our analysis we have reduced the convergence slightly between hypotheses and reduced the variance within a single hypothesis. Moreover, the central tendencies of our sampling distributions are producing far more plausible estimates of the parameter of interest (strength of selection).

There is still, however, some potentially problematic overlap between the No Preference model and the Habitat Preference model. From here, a researcher might explicitly consider the conditional probability of observing results consistent with one of these models, given the other and/or consider alternative parameterizations to assess the strrength of habitat preference that would ben necessary to reduce convergence to accpetable levels. Finally, alternative statistics/models for habitat preference could be considered that could e.g., allow greater precision in the definition of availabilty.

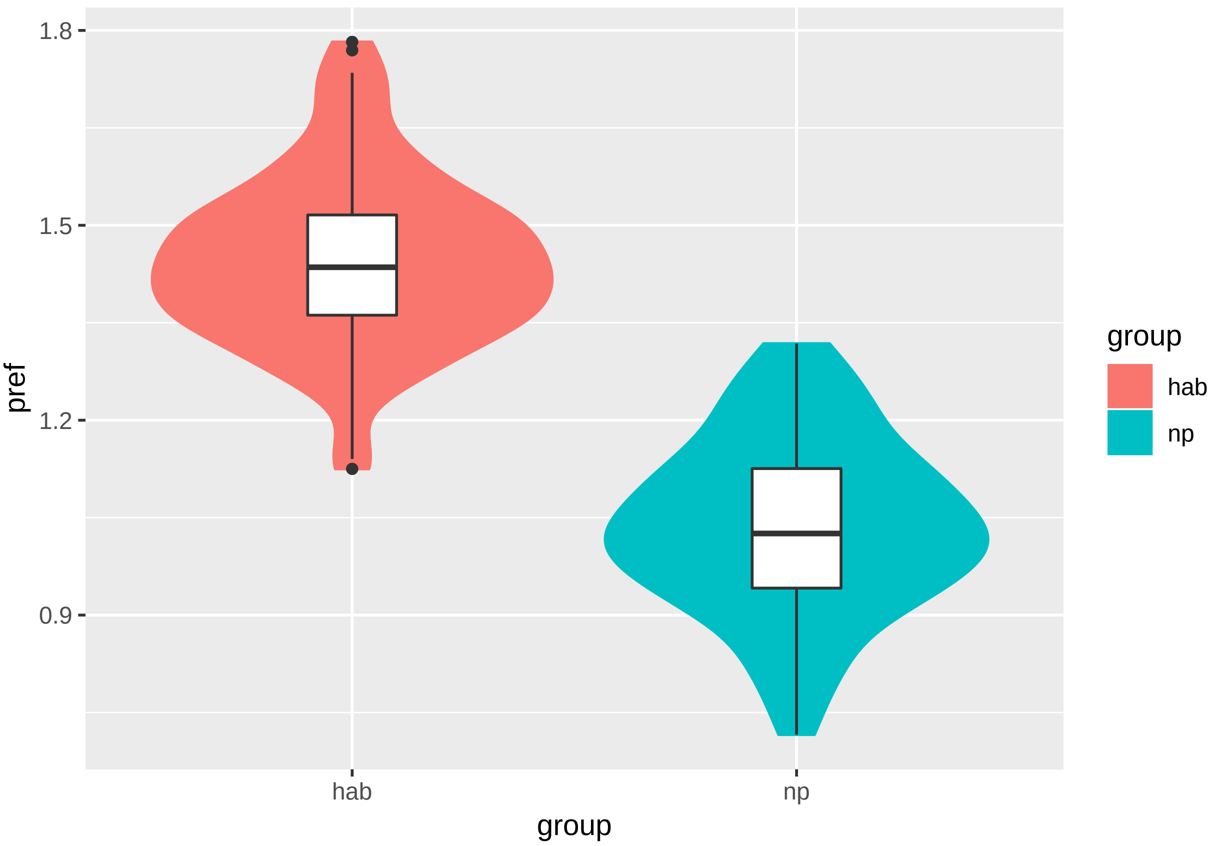

Figure 2: Filtered sampling distributions of habitat preference between models
